# ESWI pandemic preparedness summit: where science and policy meet: Tuesday 21 June 2022, Brussels and online

**DOI:** 10.1186/s42522-023-00080-7

**Published:** 2023-03-30

**Authors:** Ab Osterhaus, Colin Russell, Debora MacKenzie

**Affiliations:** 1 ESWI Chair, TiHO, Hanover, Germany; 2 ESWI Board Member, Academic Medical Center, University of Amsterdam, Amsterdam, The Netherlands; 3 Science journalist specialising in infectious disease and author of “Stopping the Next Pandemic”, Pays de Gex, France

## Keynote lectures

### The role of WHO in pandemic preparedness planning

**Dr. Maria Van Kerkhove**, *WHO, Switzerland*

The COVID-19 pandemic has not ended. In June 2022, we see 3.2 million cases and 8700 deaths recorded globally in just 1 week. This does not describe a situation of living with COVID-19 responsibly. The virus is still having a devastating effect on society and the global economy. In parallel, the risk of emergence of new or already known zoonotic pathogens is increasing due to factors such as increasing environmental degradation, rapid urbanisation, and international travel and trade.

**Fig. 1:**
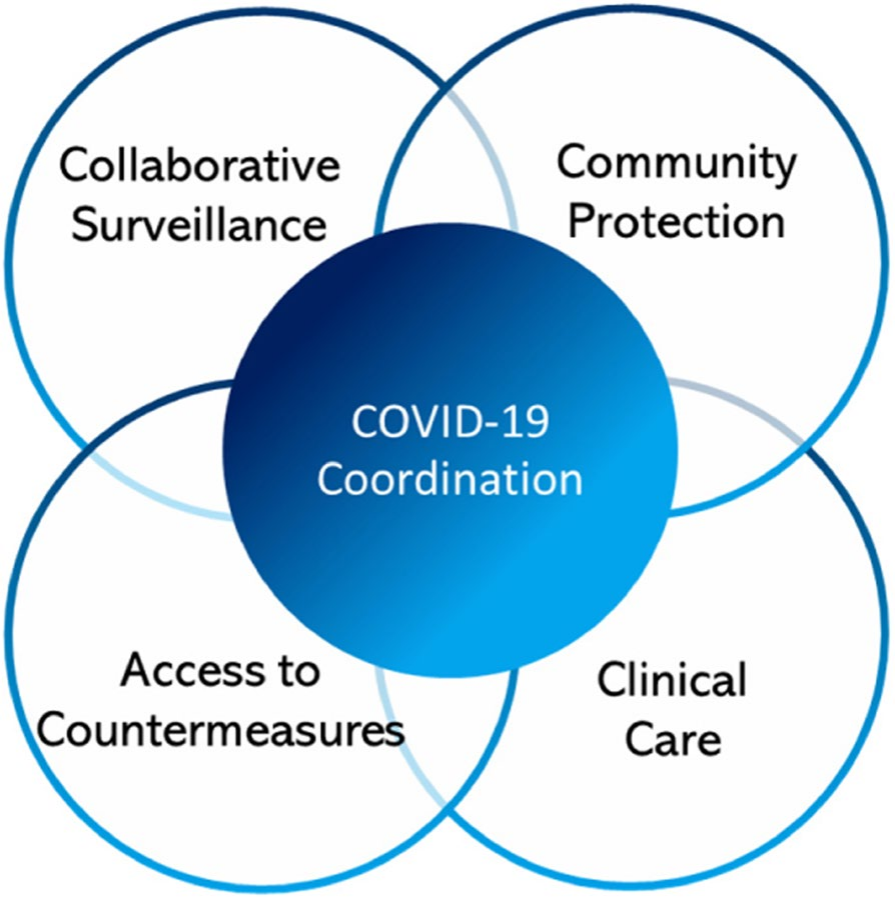
Five core components of pandemic preparedness

The WHO is working hard to end this emergency at a global level as well as to optimise international readiness for future pandemics. Indeed, everything being done now to end this emergency globally lays the basis for pandemic preparedness. Preparedness involves strengthening collaborative surveillance; community protection; clinical care; access to countermeasures; and coordination. The importance of all these measures has to be clearly and strongly communicated to politicians, economists, and the business sector (Fig. 1).

The virus will continue to evolve and circulate at different rates in different countries. However, of great concern is the sharp decline in the number of genetic sequences available to scientists throughout the world. Surveillance activities urgently need to be maintained or increased as genetic sequencing is at the heart of future vaccine development.

More than 12 billion doses of vaccines have been administered but inequities persist. Billions of people have not received the full course of vaccination, which is putting people at risk for severe disease, hospitalisation and death. A window of opportunity exists, and needs to be given political attention.

Flexible vaccination funding is essential, as well as integration of vaccination into humanitarian activities, and investing in primary healthcare systems so that vaccines can be used at the most local level. The WHO’s target is 70% of all populations in all countries. Within this overall target, specific sub-targets are 100% of all healthcare workers and 100% of all vulnerable populations.

The learnings from COVID-19 need to be used to prepare for future influenza and other respiratory disease pandemics. Respiratory disease pandemic planning and surveillance need to be integrated, along with disease management. The right architecture has to be built now. Surveillance systems need to be maintained and integrated. The workforce needs to be more agile. Trust needs to be (re)built. The most vulnerable need to be fully vaccinated. And long-term goals need to be worked towards, to develop sustainable systems for respiratory disease preparedness response.

### Future preparedness for newly emerging infectious diseases

**Prof. Zhengli Shi**, *Wuhan Institute of virology, China*

**Fig. 2:**
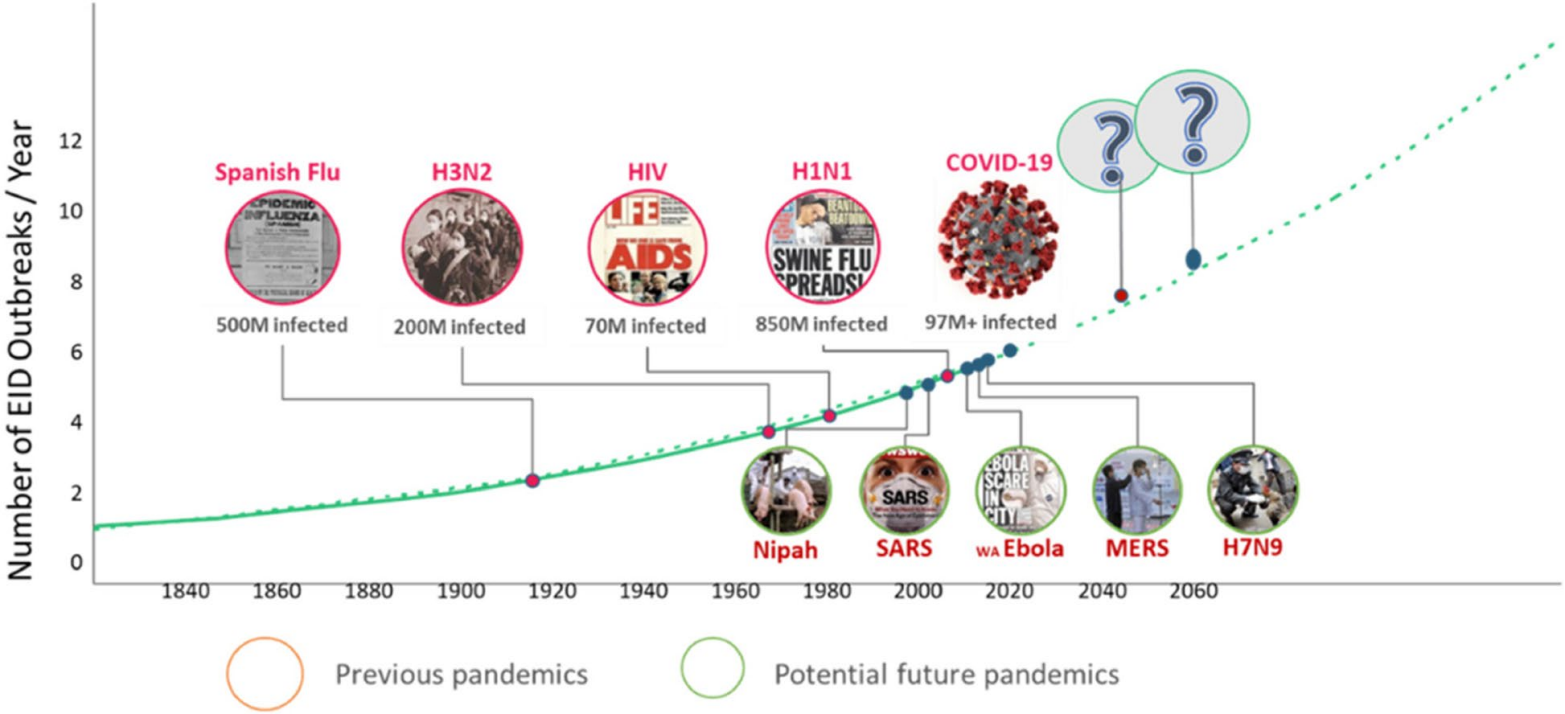
The majority of emerging infectious diseases are zoonotic, and their frequency is increasing

Infectious disease outbreaks are increasing in frequency, including of novel viruses and new strains of existing ones. The majority of emerging infectious diseases are zoonotic. Microbes are a key part of wildlife diversity, while anthropogenic environmental changes are increasing spill-over between wildlife, livestock and people (Fig. 2).

Research conducted to determine the natural reservoir of viruses discovered that bats are reservoirs for more than ten virus families, including a high proportion of coronaviruses, some of which have a high risk of jumping to other species.

To assess the potential of inter-species transmission it is important to understand the biological characteristics of novel viruses, and in particular the biological receptors. Research discovered that novel bat coronaviruses in south China use the same receptor in humans as the MERS coronavirus (DPP4) and SARS-CoV-1 and SARS-CoV-2 (ACE2). Through investigating pig farm infections it was also found that Swine Acute Diarrhoea Syndrome coronavirus (SADS-CoV) originates in local *Rhinolophus* bats, proving that bat viruses can easily jump to susceptible farm and even domestic animals. In fact, all human coronaviruses have an animal origin; mostly bats and rodents.

The question therefore arises as to how to predict and prevent newly emerging infectious diseases. This is highly challenging due to spill-over from reservoir hosts to intermediate hosts such as farm animals which are often in close contact with many humans. However, certain pre-emptive strategies are needed, such as (in surveillance and precaution) pathogen discovery, genomics characterisation, mutation & evolutionary analysis, epidemiology testing, and predictive modelling; or (in pathogen biology) looking at structure & function, entry & replication mechanisms, infection models, pathogenesis, and cross-species risk assessment; and (countermeasures) development of diagnostic methods, antivirals & antibodies, and vaccines.

In all these areas, challenges exist. These include pathogen investigation, data sharing, collaboration, transparency, and the implementation of a OneHealth approach. Unfortunately, conspiracy theories on SARS-CoV-2 origin are still prevalent, and are negatively affecting the work towards future preparedness.

### HERA: What’s new in pandemic preparedness?

**Dr. Wolfgang Philipp**, *HERA, Belgium*

HERA – the Health Emergency Preparedness and Response Authority – was created during the current pandemic with a strong legal basis for coordination and response at the EU level to health threats. It aligns with the three key aspects to pandemic preparedness: improve surveillance, strengthen healthcare systems, and increase the accessibility and availability of medical countermeasures. It specifically aims to develop local manufacturing capacities and support access to EU-funded medical countermeasures. It seeks to avoid ad hoc responses to a pandemic, by setting in place more permanent structures with adequate tools and resources. For this it has an operational budget of six billion euros from 2021 to 2027, a staff of 120, and works in two modes: a preparedness mode and a crisis mode.

Lessons learned through the COVID-19 pandemic so far were described and include the following. Better surveillance and epidemic intelligence. Overcoming the fragmented European and global surveillance ecosystem. Overcoming the data sharing challenges and lack of resources at all levels. Overcoming insufficient data quality, including missing contextual information. HERA is also working with other partners to build a stronger global surveillance system, not only to identify and report on cases but also to provide the relevant contextual information.

In R&D, HERA is funded through Horizon Europe and supports the development of next-generation vaccines and the pre-clinical development of immunotherapies. It aims to create a common and strategic research and innovation agenda for pandemic preparedness; something that needs to stay at the top of the international agenda. In this respect HERA supports the functioning of the Global Research Collaboration for Infectious Disease Preparedness.

**Fig. 3:**
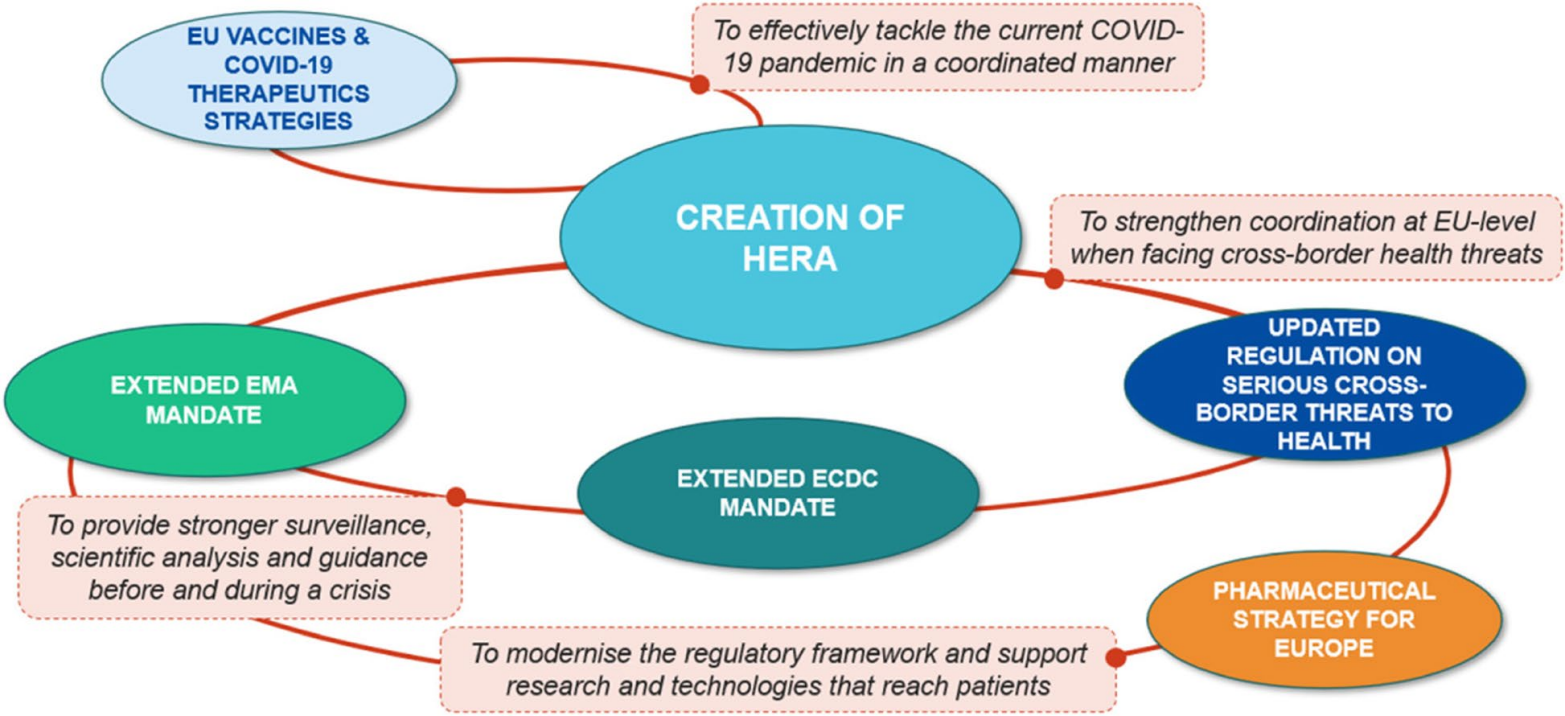
HERA aims to reinforce the global health emergency preparedness and response architecture

When it comes to addressing the market failures during this pandemic, HERA is working to make critical supply chains more resilient; here it is vital for the health sector to identify what is critical and what is not. A Commission taskforce was created to address supply chain bottlenecks and make some of them more accessible. Critical vaccine production must be ensured. It is also essential to kick-start an immediate response to produce medical countermeasures when they are needed. HERA is investing in a strategic stockpile of products, from antibiotics to PPE. Clinical trials is another area of investment and a clinical trials network has been set up. Strengthening knowledge and skills is also key to make sure that a certain benchmark is reached when it comes to aspects such as procurement and the necessary collaborations (Fig. 3).

HERA focuses on having an inclusive approach with Member States incorporating science, academia, industry and civil society, to provide a sustainably financed instrument or organisation that takes care to ensure access to medical countermeasures. Thanks to all these and other activities, the EU is better prepared for the next pandemic.

## Keynote lectures

### Options for global and accessible vaccines

**Dr. Rino Rappuoli**, *GSK, Italy*

During the COVID-19 pandemic, vaccines were produced in only 10 months. This was possible by the combination of new technologies and significant public health sector investment. Four technologies in particular accelerated vaccine development: Internet-based vaccines, structural biology, synthetic biology, and adjuvants.

Internet-based vaccines development involves shipping the necessary sequencing information on a virus over the internet rather than shipping actual viruses. This transition – from analogue to digitally shared vaccine development – will be how vaccines can be made available globally in the future. A sequence will be sent via the internet, a computer will analyse the information and then design a vaccine. The information on this vaccine can then be transmitted over the internet to a number of robotic stations located remotely through the world where the vaccine can be made locally. This is an easier and quicker process than the traditional methods to make vaccines.

The other important contribution to make vaccines is the investment. Today, making a vaccine needs an investment of at least 1 billion dollars: 10% in the discovery phase, 20% in early development and 70% in late development, manufacturing and registration. Usually, investment in early development does not take place unless discovery is a success. Likewise, investment in late development depends on the success of early development. All of this takes time as it happens sequentially.

When COVID-19 arrived, the public sector mostly invested the money in different companies working on these different phases in parallel. In addition, companies were encouraged to move quickly to the next phase as soon as they had results, without waiting for that phase to come to completion. This different approach was a huge revolution that allowed the whole vaccine development process to move much faster.

**Fig. 4:**
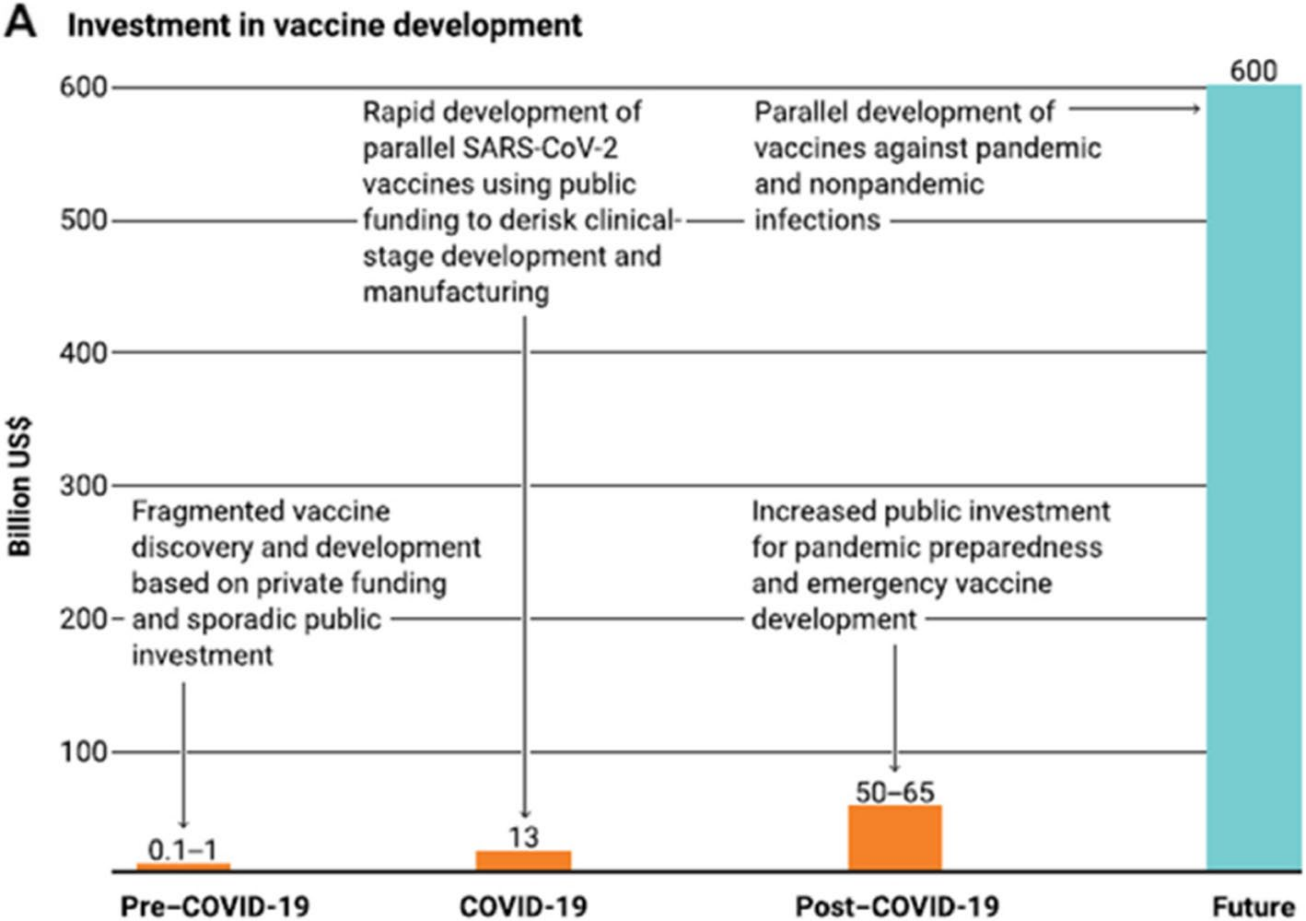
The trillion dollar vaccine gap

The lesson is that investment is vital. Interestingly, the investment of 12 billion dollars that was made in COVID-19 vaccines was minimal compared to the global economic losses of 500 billion dollars per month due to the pandemic. To ensure proper preparedness, an investment in vaccines of one trillion dollars is necessary. Having experienced the huge global economic and health disruption of the pandemic, such investment is vital to prevent this happening again in the future (Fig. 4).

### International health regulations: making them work better

**Prof. David Heymann**, *Chatham House, United Kingdom*

The initial International Health Regulations (IHR) created in 1969 aimed to maximise security against the international spread of infectious diseases with minimal interruption of travel and trade. They involved notifying the WHO of an occurrence of cholera, plague, yellow fever or smallpox. They also set out health measures that a country may require for protection, along with the appropriate health organisation at borders to prevent vector proliferation. The WHO distributed a weekly bulletin, and a country could then put into place the necessary countermeasures.

However, not all countries reported infections under the IHR, some in an effort to avoid sanctions against them which would affect their economy. At the same time, many new diseases were emerging, for which the IHR were not particularly useful.

**Fig. 5:**
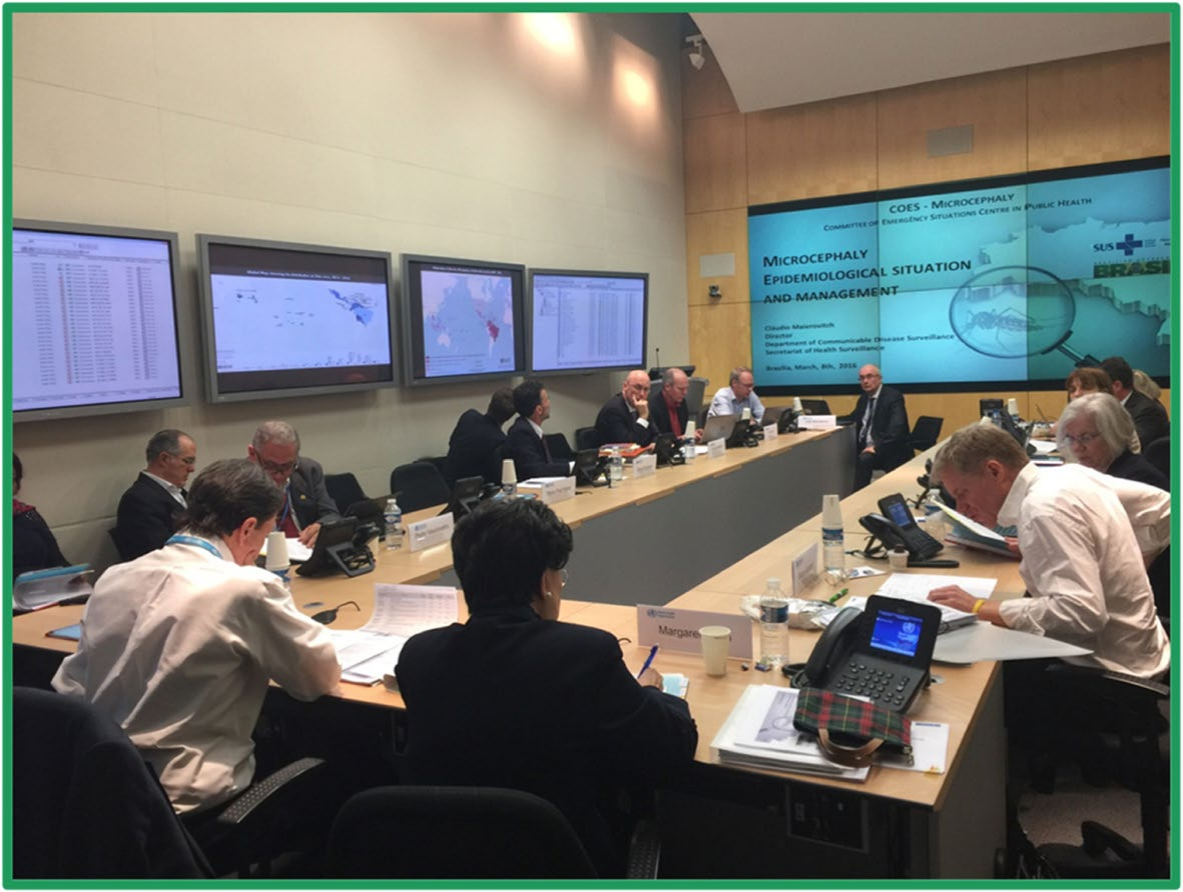
The IHR Emergency Committee at work assessing a Public Health Emergency of International Concern (PHEIC)

WHO was therefore asked by the World Health Assembly to revise the IHR in 1996 to take into account up-to-date global communication and collaboration, and to change the norms surrounding reporting of infectious disease outbreaks. The Global Outbreak Alert and Response Network (GOARN) was set up as part of the revision process, which proved useful to manage the SARS outbreak in 2003. In 2004 the revision process was intensified, and the final revision in 2005 encompasses all public health threats; moves from passive to proactive surveillance; and importantly moves away from control at borders to detection and containment at source. A decision-tree analysis now determines if an occurrence is potentially of international public health importance, and if so an emergency committee meeting is called. This can lead to a decision by the Director General to announce that the event is a public health emergency of international importance (PHEIC), and to risk-based public health measures being recommended proactively by the WHO (Fig. 5).

During the COVID-19 pandemic, many although not all of the WHO’s recommendations were followed after the announcement of a PHEIC. A notable exception was that WHO member countries decided to adopt travel recommendations based on the risk assessment of national advisory groups, not on the WHO’s initial blanket recommendation to not adopt barriers to international travel. This was contrary to previous recent public health events when WHO was accepted as the major source of information and guidance. One reason could be the abundance of scientific evidence on COVID-19 available on the Internet.

Consequently, questions arise as to the functions and scope of the IHR for future pandemic preparedness. Are they a vestige of the twentieth century? Do they clearly define data sharing? Do they provide sharing of benefits of public health innovations? Do they take advantage of the support that can be provided by the private sector? Is there a need for a standard methodology to assess the risks and benefits of closing international borders to traffic with the aim of delaying virus introduction? Will a pandemic treaty compensate for the weakness of the IHR, or will there be another revision?

Moving forward, perhaps a pandemic treaty that enshrines much of what the IHR aims to do could strengthen the IHR so that they become a widely-accepted framework for risk assessment. This will depend on consensus developed during the forthcoming global discussions on a pandemic treaty.

### Translating science to policymakers and the public at large

**Prof. Christian Drosten**, *Charité Universitätsmedizin Berlin, Germany*

When communicating to the public, science and scientists need to bear in mind certain responsibilities: Seeking opportunity rather than opportunism; Enabling and facilitating access to research resources for other disciplines; Prioritising immediate outcome vs. long-term success and sustainability; Avoiding overpromising; And maximising assistance and service to society during a crisis.

A scientist also has a responsibility to be authentic towards politicians, and not to mix advice and opinion. Limits of knowledge need to be carefully considered and communicated, and conflicts of interest avoided. Accountability has to be maintained by documenting advice given and delimiting responsibilities.

Towards the media, a scientist needs to support freedom of information and avoid manipulation of reporting and one-sided communication. Towards society, it is necessary to be accountable towards the tax payer and provide information as part of a package of non-pharmaceutical intervention.

**Fig. 6:**
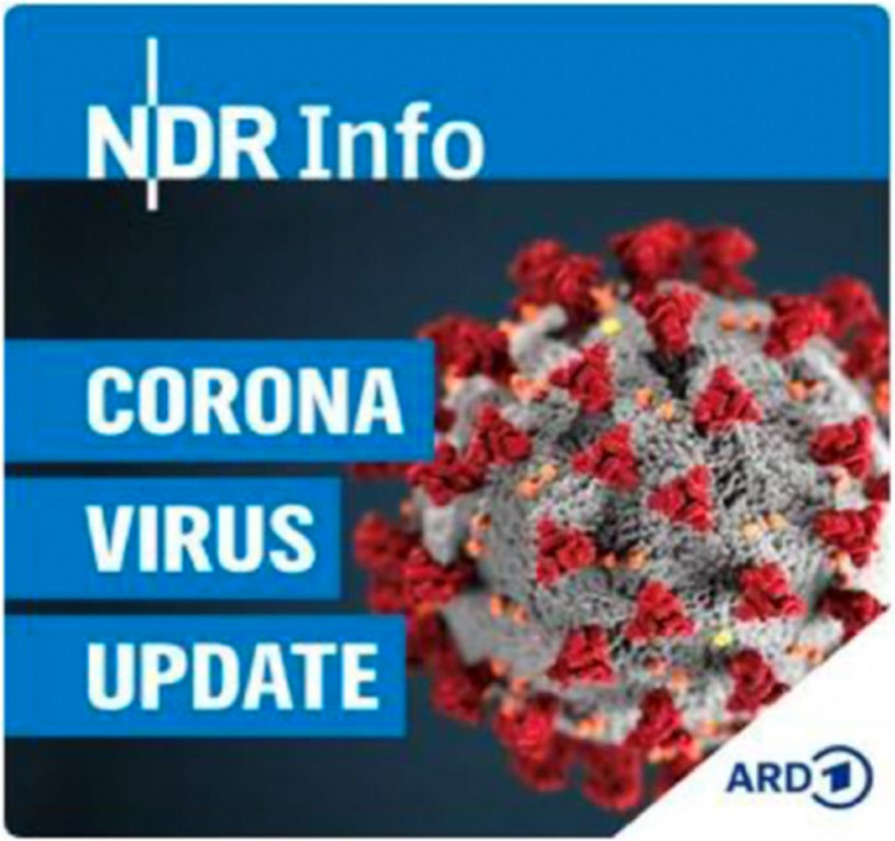
The German-language Coronavirus update podcast was accessed over 142 million times, and 75% of listeners reached the end of each edition

Through the pandemic, a coronavirus update podcast run by Prof Drosten in Germany was accessed over 142 million times. It was provided only in German and a number of scientific and medical speakers were featured on the podcast, which also included science journalists (Fig. 6).

Public perception in Germany during the pandemic went in phases, from the calm before the storm through the various waves, recoveries & returns, and the arrival of further strains up to Omicron and the present situation. Throughout each phase there were always two main prevailing opinions or sides, along with their public messaging, which were described in detail.

Scientific communicators face continuing challenges. These include growing attacks from para-science and some media sources, plus a loss of credibility with not-so-well informed politicians. There is a loss of voice due to public attention refocusing on the war in Europe, and a loss of courage, optimism, funding and support. The growing immunisation rate confirms mis-assessments, (“it’s becoming like the flu”), which gives confirmation to those who always claimed that COVID-19 was just the flu. This is leading to the appearance of retrospective strawman narratives on schools, lockdowns etc.

To conclude, population-level infection control can only work with well-informed citizens. Public information is one of the prime interventions, especially during the early phase of a pandemic. Quality control in scientific communities must urgently be extended to include science communication. Journalism must urgently re-consider its responsibilities in controlling the quality of public messaging.

### The role of scientists in advising policymakers in a pandemic. What do we do now? How do we make it work?

**Prof. David Fisman**, *University of Toronto’s Dalla Lana School of Public Health, Canada*

**Fig. 7:**
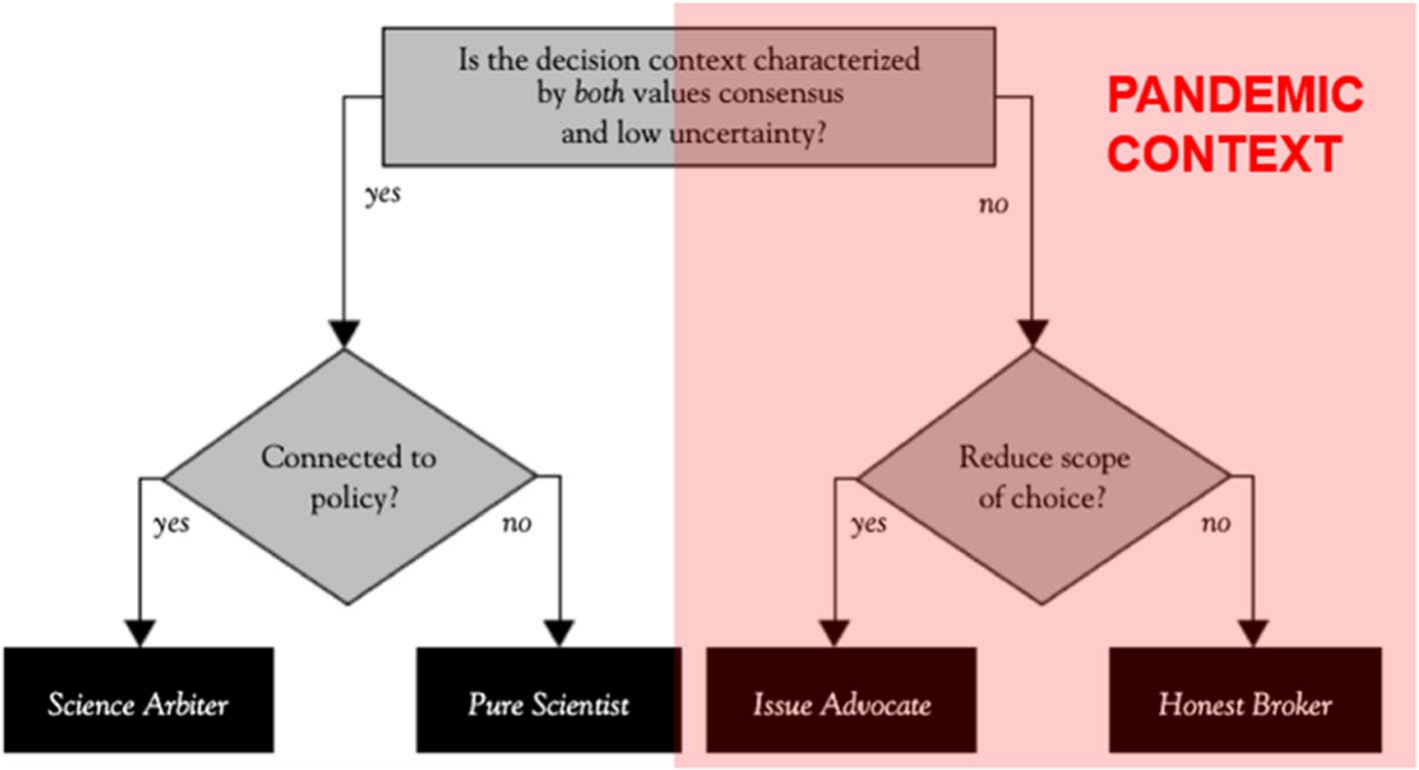
Criteria for determining the roles of science in policy and politics

According to Roger Pielke in The Honest Broker, the scientific community has four possible roles to play: science arbiter, pure scientist, issue advocate or honest broker. The dividing line is that it is possible to be a pure scientist or a science arbiter when it is a simple decision, when values are shared in terms of the desirable outcome, and where there is little uncertainty in terms of what the data show. However, in a pandemic there is likely to be a lot of uncertainty and few shared values, so the only two possible roles are issue advocate or honest broker (Fig. 7).

The tough decisions that need to be made during a pandemic share some characteristics. The scope of choice can be ambiguous or continuous. Conflicts exist around the desirability of different outcomes. Ambiguity exists around conditions that motivate the need for decision-making. More information provides little insight into courses of action likely to lead to a desired outcome. Uncertainty is a resource for various interests during the process of bargaining, negotiation and compromise.

Some elements of the pandemic were well understood early on, for example where sufficient data existed to allow implementation of control. Other elements were understood later and were perhaps harder to predict. Examples of the former are the dominance of aerosol dispersion and the importance of masking, while the rapidity of vaccine development belongs to the latter.

Casual reading of newspapers or consulting media sources may suggest significant scientific uncertainty, although in reality this was probably less than suggested. The lack of both coherence and consistency is a result of how outcomes are valued. Obviously, multiple potential objectives were targeted in parallel, such as preventing deaths, preserving health systems, maintaining mental health, maintaining educational systems, protecting economies etc. Some of these have been more difficult for people to articulate out loud or present as their value or goal. This gives rise to the “manufacture of uncertainty” via well-funded disinformation campaigns. In other words, in situations where science can’t be argued, it is easier to manufacture uncertainty.

A further challenge is that the world is currently in a very difficult political environment, which provides fertile soil for pandemic disinformation that contaminates the political culture (certainly in Canada but also elsewhere).

Looking to the future, the world is in the era of pandemics for well-known reasons. Ambiguity and (manufactured) uncertainty leave only the roles of issue advocate and honest broker. The informational environment we now live in, with the ease of creation and dissemination of disinformation, and the manipulation of populations, needs to be considered as a future component of pandemic threats.

## Expert panel views on preparedness

**Prof. Linfa Wang**, *Duke-NUS Medical school, Singapore*

Pandemic preparedness goes together with pandemic response. Dealing with a potential pandemic can be divided into three stages. The first stage is the animal-human interface, because most pandemics in the last 20 years are zoonotic in nature. The second stage is the early warning; where a very unusual case or a cluster is identified. This is the most difficult stage. Questions need to be asked as to whether the reporting system in place in early 2020 was adequate. Early warning depends on having a very clear picture that one pathogen is causing an outbreak, and it is up to the country to report it. But it is interesting to note that the first evidence of a coronavirus in December 2019 came from doctors, not scientists. Front-line clinicians could not understand this “severe pneumonia” so they sent samples to private sequencing companies who identified a bat coronavirus.

How can the reporting system be changed? Instead of a system reporting a *confirmed* etiology, can a system be put in place that reports *suspected* etiology? This could become available 3–4 weeks earlier than the clinical confirmation.

The third stage involves the rapid development of countermeasures. It was an excellent achievement to get vaccines in 10 months. However, the world is still experiencing the pandemic; vaccination has not contained it as totally as we would have wished.

In short, the success of pandemic preparedness will depend heavily on transparent and effective international collaboration; a sustainable funding model; and an emphasis on basic research during peacetime.

**Dr. Lwazi Manzi**, *AU Commission on Africa’s COVID-19 Response, South Africa*

Pandemic preparedness is required at community, national, regional and global levels. The African Pandemic Preparedness and Response Authority (APPRA) derives its mandate from the Africa Union Heads of State and Government. It aims to develop a continental pandemic preparedness and response mechanism that can address critical issues related to coordination, financing and equitable access to medical countermeasures.

It is necessary because existing global systems such as WHO and IHR failed the continent during HIV-AIDS, Ebola and COVID-19. Another example is the current monkeypox epidemic. Africa has recorded over 10,000 cases and 600 deaths. But it was not until it spilled over into the rest of the world that medical countermeasures were mobilised. So while the AU is appreciative of multilateral arrangements and donors, at the end of the day Africa needs its own pandemic preparedness system.

The APPRA will not contradict the existing structure and mandate of the Africa CDC but will strengthen its work by providing legal mandates such as operations and declaring public health emergencies of regional concern. It will legally bind member states to a set of rules and to a playbook and protect Africa from market and global failures. It will also facilitate regional and cross-border collaborations.

The G20 is currently establishing a financial intermediary fund to finance global pandemic preparedness. Two serious concerns are that it’s G20 led, which raises questions as to the inclusion of non-G20 countries. And the voices of the funding donors are dominating the governing council and the control of the dispersion of the funds.

**Dr. Aeron Hurt**, *Roche, Switzerland*

The International Federation of Pharmaceutical Manufacturers & Associations (IFPMA) has prepared a report identifying ten industry-wide insights into future pandemic preparedness:

- Health security starts with pathogen surveillance and sharing
- Partnerships accelerate R&D and manufacturing
- Advanced market commitments support manufacturing scale-up for global pandemic response
- Innovation is essential for preparedness and response
- Global upstream supply chain disruptions put production and distribution at risk
- An established procurement mechanism for low-income countries is vital
- Regulatory agility and convergence guard safety and speed of access
- Vaccine nationalism puts everyone at risk
- Delivery infrastructure must be strengthened
- Confidence in vaccines and therapeutics is critical for success.

How could these insights be put into practice in the influenza antiviral space in the face of a potential new influenza pandemic? Antiviral agents against influenza are already available. However, the manufacturing timelines mean that demand will exceed available supply in a pandemic. There is therefore a commitment to provide stocks to WHO, to work with WHO and other NGOs on solutions for low- and middle-income countries, and to partner with governments to find long-term, sustainable solutions to enhance local pandemic preparedness. Integrated diagnostic, therapeutic and health system infrastructure approaches are needed, and innovative stockpiling approaches need to be explored.

**Dr. Mark Eccleston-Turner**, *King’s College London, United Kingdom*

The global health governance system is broken. It embeds requirements that low- and middle-income countries – where most novel and infectious diseases emerge – must put in place capacities such as surveillance mechanisms and healthcare systems to detect a novel outbreak rapidly. These obligations were put in place by the international community without any commitment to finance them. Or any recognition that low- and middle-income countries have many other priorities for their limited healthcare budgets.

Moreover, while the samples, data and information of low- and middle-income countries are considered as public goods that must be shared for the good of humanity, on the other hand, vaccines and other medical countermeasures are considered to be private goods to be horded and accessed by the world’s wealthiest first. This situation is inherently unfair.

An EU-led initiative for a new Pandemic Treaty has been proposed and is being drafted by the WHO, along the norms of solidarity, fairness, transparency, inclusiveness and equity. However, it appears that the treaty will take forward with it the same neo-colonial thinking that is embedded in the current system.

If humanity is to be prepared for the next pandemic we must fix these deep-rooted structural inequalities which are embedded in the global health system. The pandemic treaty is an opportunity to do this at a multilateral level. However, on the basis of the present proposals and the manner in which the treaty is being developed it’s clear that the treaty will fall far short of these expectations.

**Dr. Anjana Ahuja**, *Financial Times, United Kingdom*

When communicating in a pandemic it is essential to be the signal in a world full of noise – a world in which there is vast amounts of speculation, rumour and conspiracy. It is vital to give readers the tools to allow them to make sense of a fast-moving crisis situation. This means being clear, transparent and trustworthy.

In a crisis such as the recent pandemic, people want information so that they can make sense of the world around them, judge risk to themselves and their families, understand decisions made in their name by governments or others, and make their own personal decisions based on current knowledge. This information has to come from somewhere, and the information ecosystem is huge. It includes traditional media, online media, social media, official channels, and word-of-mouth.

There are many ways in which scientists and experts can help journalists communicate well in a pandemic. By being responsive to media requests and open to questions. By answering all questions asked, even the obvious ones. By being clear about what is known and unknown. And by explaining why some aspects are unknown and what further data are needed.

What doesn’t help? Anything that breaks the rules of clarity, transparency and trust, or that leaves an information vacuum. This could be because scientists may think it is too complicated or uncertain to explain, or the data is available but is not being shared. Or there could be scientific disagreement, or the information might change later on. However, in a pandemic, people want and need to know. And if they can not get it from reputable sources, they will keep searching for answers and get it elsewhere.

## Stakeholder debate

- Prof. Linfa Wang, *Duke-NUS Medical school, Singapore*
- Dr. Lwazi Manzi, *AU Commission on Africa’s COVID-19 Response, South Africa*
- Dr. Aeron Hurt, *Roche, Switzerland*
- Dr. Mark Eccleston-Turner, *King’s College London, United Kingdom*
- Dr. Anjana Ahuja, *Financial Times, United Kingdom*
- Prof. Christian Drosten, *Charité Universitätsmedizin Berlin, Germany*
- Prof. David Fisman, *University of Toronto’s Dalla Lana School of Public Health, Canada*

### What can be done to improve the equitable distribution of vaccines?

Dr. Mark Eccleston-Turner: We need to treat the information to make a vaccine in the same way that we treat the genetic sequencing and epidemiological data. Make it all open source.

Dr. Lwazi Manzi: We need a legal, binding agreement that compels member states to deploy and mobilise the necessary finances and resources to where they are urgently needed.

Prof. Christian Drosten: It is difficult for countries to determine vaccine effectiveness in a country if a suitable surveillance system is lacking. Detection has to be facilitated at a technical level first. For that, the IHR provides a sufficient legal framework.

Prof. David Fisman: We have to start with our values rather than information. Is equity something we really value? If so, then global financing needs to be made available now.

Dr. Anjana Ahuja: The pandemic treaty binds poorer countries but does not protect them. At the same time, it protects richer countries but doesn’t bind them. This means inequity is built-in.

Dr. Aeron Hurt: Preparedness is about addressing the challenges and overcoming the hurdles during peacetime with simulations and by pressure-testing. We will then know what we can rely on and what needs changing.

Prof. Linfa Wang: Military preparedness is in the DNA of every nation and takes up a huge percentage of a country’s GDP. Can we raise our pandemic preparedness to the military preparedness level?

Dr. Mark Eccleston-Turner: Why do we need to have intellectual property? It remains an imperfect system that does not meet the needs of vast swathes of the developing world. The pharmaceutical industry argues that they need 20 years to recoup their investment. But for COVID-19 that doesn’t ring true. Much of the upfront investments were made by governments – who then paid for the vaccines that they paid to develop. We are socialising risk and privatising profit. The IP system doesn’t work. We can do better.

Mrs. Debora MacKenzie: Looking back, the vaccine used in the 1960s and 1970s to eradicate smallpox was largely produced by publicly-owned labs in the Soviet Union, the US and other countries, as a public good. With market forces now hampering the development of vital drugs such as new antibiotics, among others, it is time more medicines were again handled as public goods. That means the IP system must be re-drawn so it protects both public goods and private profits in ways that do not undermine public health.

### How can we improve the early detection and early warning system?

Dr. Lwazi Manzi: We need to have the ability to declare a public health emergency of regional concern. We need a mechanism to expedite the reporting of a suspected outbreak and the appropriate response without having to wait for a global mechanism that takes too long to spring into action.

Dr. Anjana Ahuja: The defence community is really good at spotting the signal above the noise. This is what is needed in disease surveillance. Pandemic threats should be regarded as security threats.

Mrs. Debora MacKenzie: Many countries cover up disease. We need better surveillance everywhere, detecting and reporting outbreaks, then international inspectors empowered to go into any country to observe any outbreak first-hand, and report it publicly – just as we now have for chemical and nuclear weapons . When risk is global, responsibility must also be global, not entirely subject to national sovereignty.

Dr. Mark Eccleston-Turner: At the same time there has to be response in terms of human, financial and medical countermeasures to help that country contain an outbreak instead of being punished with trade and travel restrictions.

Prof. Christian Drosten: In the global south, capacity building, training, empowerment, and the creation of an academic environment is vital. Yet in many countries in Africa it’s virtually impossible to develop a research career. As long as this is not understood by local governments, the brain drain out of Africa will continue.

### In Europe there were 27 (28 with the UK) different policies; sometimes contrary. What’s the solution to this?

Prof. David Fisman: It is not possible to put binding regulations in place across all of Europe because the demography, the epidemiology and the surveillance systems vary so widely. But it could be useful to set a standard reference for basic traits of the infection, such as transmissibility with age or social situations, or the effectiveness of non-pharmaceutical interventions.

Dr. Anjana Ahuja: Every country should not have the same blueprint. Each government has a social contract with its citizens, and the decisions rest with that government when it comes to imposing potentially binding regulations.

### What could be done to improve communication?

Dr. Lwazi Manzi: We could use more and varied communicators to communicate the information available and to counteract the vast disinformation on social media.

Prof. Christian Drosten: Public health messaging and communication to citizens during a pandemic needs quality control.

### What would be your single takeaway or final thought?

Dr. Anjana Ahuja: I am constantly amazed at how people do things that are so obviously against their self-interest. We’re in a communication battle.

Dr. Lwazi Manzi: Our heads of state have issued a mandate to strengthen regional capacity and we ask that all our partners align with this mandate and work together with us to come up with a win-win solution in terms of vaccine procurement and manufacturing on the African continent.

Dr. Aeron Hurt: We should not forget some of the wins that have been achieved – in speed of response with some medical countermeasures, for example – and take those forward to further strengthen the ability to provide these medicines to where they are needed the most.

Dr. Mark Eccleston-Turner: There is huge structural inequality in global health, with a long history of colonial exploitation. The current system is about protecting “us” from threats that occur “over there.” The global health governance architecture is broken and needs to be fixed.

Prof. David Fisman: Ventilation, filtration, and upper-room UV germicidal irradiation and other means to manipulate indoor environments are likely to be fundamental to dealing with future respiratory disease pandemics. Just like water sanitation was vital to get rid of cholera.

Prof. Linfa Wang: A pandemic is not just a public health issue but a security issue. We can prepare for a war or a terrorist attack; it is time to bring pandemic preparedness up to that level.

Prof. Christian Drosten: We need a much better interface between science and policymaking, which could involve more scientists becoming involved in policy. Finally, we could be watching a new pandemic in the making, with monkeypox, and we need to take the threat much more seriously than we are doing at present.

